# Supplementary material for: Evolution of triclosan resistance modulates bacterial permissiveness to multidrug resistance plasmids and phages
Source: Nat Commun. 2024 Apr 30;15:3654. doi: 10.1038/s41467-024-48006-9 (PMC11061290; doi:10.1038/s41467-024-48006-9)
Supplement: Supplementary file 1 — Supplementary Information [file 41467_2024_48006_MOESM1_ESM.pdf]

Supplementary data for:

Evolution of triclosan resistance modulates bacterial permissiveness to multidrug resistance plasmids and phages

Qiu E. Yang[1], Xiaodan Ma[1], Minchun Li[1], Mengshi Zhao[2], Lingshuang Zeng[1], Mingzhen He[1], Hui Deng[2], Hanpeng Liao[1], Christopher Rensing[1], Ville-Petri Friman[3], Shungui Zhou[1], Timothy R. Walsh[4]

Supplementary Table S1-S6

Table S1 The list of strains and plasmids used in this study

Strain ID	Species	Antibiotic resistance	Origin	References
		**Strains**		
KP85anc	*K. Pneumoniae*	Tig^R	Patient feces	Ref [1]
d7-1	*K. Pneumoniae*	Tig^R	evolved clone(day7)	This study
d7-2	*K. Pneumoniae*	Tig^R	evolved clone(day7)	This study
d7-3	*K. Pneumoniae*	Tig^R	evolved clone(day7)	This study
d7-4	*K. Pneumoniae*	Tig^R	evolved clone(day7)	This study
d7-5	*K. Pneumoniae*	Tig^R	evolved clone(day7)	This study
d10-2	*K. Pneumoniae*	Tig^R	evolved clone(day10)	This study
d10-3	*K. Pneumoniae*	Tig^R	evolved clone(day10)	This study
DH5α	*E. coli*	Amp^R	Laboratory strain	NEB, #C2987
MG1655	*E. coli*	Kan^R	Laboratory strain	
		**Plasmids**		
pX3_NDM-5	IncX3	Mero^R	Wild type plasmid	Ref [2]
pFII_MCR-8	IncFIIk	Coli^R	Wild type plasmid	Ref [3]
pX4_MCR-1	IncX4	Coli^R	Wild type plasmid	Ref [4]
pA/C_MCR-8	IncA/C	Coli^R	Wild type plasmid	Ref [3]
pHSG299	cloning vector	Kan^R	Lab plasmid	
pCasKP	CRISPR-Cas Plasmid	Apr^R	Lab plasmid	Ref [5]
pSGKP-spe	CRISPR-Cas Plasmid	Rif^R	Lab plasmid	Ref[5] (purchased from addgene# 117234)

Table S2 The list of primers used in this study

**Primers**	**Sequence (5'-3')**	**Size (bp)**	**Application**
ndh-F	GAAAGCGAAAATCACCCTGG	750	PCR for *ndh* amplicon
ndh-R	CATGAAATCTGGCGCTTTGA		
nsrR-F	TGGTTCCGCTAACTCGTGAC	290	PCR for *nsrR* amplicon
nsrR-R	GGTATCGGCGTTTTTGACCG		
mcr-1-F	GCTACTGATCACCACGCTGT	980	PCR for *mcr-1* amplicon
mcr-1-R	TGGCAGCGACAAAGTCATCT		
mcr-8-F	TTCTACAAAGCGTGGGGGAG	713	PCR for *mcr-8* amplicon
mcr-8-R	CATAGCAACGTCACGCATCC		
NDM-5-F	AACGGTTTGGCGATCTGGTT	540	PCR for NDM-5 amplicon
NDM-5-R	GTGTCGGCATCACCGAGATT		
pCas-kp-F	CCGTCGTTGGAACTGCTTTG	670	PCR Verification for pCas-kp
pCas-kp-R	AGCATCCGTTTACGACCGTT		
299-homo-F	CCGCTTACCGGATACCTGTC	921	PCR Verification for pHSG299
299-homo-R	TCGCGAGCCCATTTATACCC		
p299-ndh-F1	GCTCAGTGGAACTCCGTCGATTAGTGCAGCTTCAGGCG	~1500	PCR for *ndh* amplicon with pHSG299-overlapped length
p299-ndh-R1	TTCTTCACGAGGCAGACCTCTGGTATGACCAATGCACC		
p299-nsrR-F2	GCTCAGTGGAACTCCGTCGACCTTTGCCCGTGAAATCAG	~700	PCR for *nsrR* amplicon with pHSG299-overlapped length
p299-nsrR-R2	TTCTTCACGAGGCAGACCTCTTAATGCGCTTTTTTAAAGCG		
nsrR-299-homo-F	AGCTCTTGATCCGGCAAACA	~1700	PCR Verification for pHSG299:*ndh*
nsrR-299-homo-R	TCAGATCACGCATCTTCCCG		
pSGKP-ndh-N20F1	CGACCACCAGCTGATTGATAGTTTTAGAGCTAGAAATAGCAAGTTAAAATAAGGC	~4500	*ndh*-Spacer construction
pSGKP-ndh-N20R1	TATCAATCAGCTGGTGGTCG ACTAGTATTATACCTAGGACTGAGCTAGC		
pSGKP-nsrR-N20F1	ACAGCTTTGCAGATGGTTCT GTTTTAGAGCTAGAAATAGCAAGTTAAAATAAGGC	~4500	*nsrR*-Spacer construction
pSGKP-nsrR-N20R1	AGAACCATCTGCAAAGCTGT ACTAGTATTATACCTAGGACTGAGCTAGC		
ssDNA-ndh	GAAGAGCCCGGAGGCGCCCTCAGAGCGCGCCTCCGGGTCGCGACAGCGATATTTCAATAACTTTTATTTAACAATTGGTTAATAATTTTAGGGGTACAC	90	Gene deletion
ssDNA-nsrR	CTCCGCGGTGCCAAAAAGAACAAGATTCACCGCAACCCAGGACACTGCCACCGCCTTCACAAGTTGAAGGCGGTTTTTTTCGTCACCAGAAAATGA		Gene deletion
p299-F	GAGGTCTGCCTCGTGAAG	2300	PCR for full-length pHSG299
p299-R	TCGACGGAGTTCCACTGAG		
pSGkp-N20-F	CAAACCCGCGCGATTTACTT	497	PCR for spacer verification
pSGkp-N20-F	TGTGTGGAATTGTGAGCGGA		

Table S3 A number of survival populations over 11-day evolutionary course

Replicates	day 0	day 6	day 7	day 8	day 9	day 10	day 11
TCS(mg/l)	0	1 (2xMIC)	2 (4xMIC)	4 (8xMIC)	8 (16xMIC)	16 (32xMIC)	32 (32xMIC)
replicate-1	d0-1	d6-1	d7-1	d8-1	×	×	×
replicate-2	d0-2	d6-2	d7-2	d8-2	d9-2	d10-2	×
replicate-3	d0-3	d6-3	d7-3	d8-3	d9-3	d10-3	×
replicate-4	d0-4	d6-4	d7-4	d8-4	×	×	×
replicate-5	d0-5	d6-5	d7-5	×	×	×	×
replicate-6	d0-6	d6-6	d7-6	×	×	×	×
replicate-7	d0-7	d6-7	d7-7	×	×	×	×
replicate-8	d0-8	d6-8	d7-8	×	×	×	×
replicate-9	d0-9	d6-9	×	×	×	×	×
replicate-10	d0-10	d6-10	×	×	×	×	×
replicate-11	d0-11	d6-11	×	×	×	×	×
replicate-12	d0-12	×	×	×	×	×	×
replicate-13	d0-13	×	×	×	×	×	×
replicate-14	d0-14	×	×	×	×	×	×
replicate-15	d0-15	×	×	×	×	×	×
**Survival No.**	**15**	**11**	**8**	**4**	**2**	**2**	**0**

*note: evolved strains highlighted in red indicate those strains were isolated and sequenced; “x” indicates no survival bacterial in this replicate, which was also highlighted in grey.

Table S4 Antibiotic susceptibility of ancestral and evolved Kp85 clones.

Stain ID	TCS*	CIP	FOS	TIG	TET	CHL	CTZ	MERO	CTX	COL
Kp85anc	0.25	0.5	8	32	>128	128	0.25	0.06	0.25	2
d7-1	8	4	32	64	>128	>128	1	0.06	0.25	2
d7-2	8	8	32	32	>128	>128	0.5	0.06	0.25	2
d7-3	4	8	64	32	>128	>128	0.25	0.06	0.25	2
d7-4	4	16	32	32	>128	>128	0.25	0.06	0.25	2
d7-5	8	8	128	32	>128	>128	0.25	0.06	2	2
d8-1	16	4	128	64	>128	>128	0.25	0.06	2	2
d8-2	8	16	123	32	>128	>128	0.25	0.06	2	2
d8-3	4	4	32	32	>128	>128	0.25	0.06	0.5	2
d8-4	4	16	32	32	>128	>128	1	0.06	0.25	2
d9-1	32	8	64	32	>128	>128	0.25	0.06	0.5	4
d9-3	16	8	64	32	>128	>128	0.25	0.06	0.5	2
d10-2	32	8	256	32	>128	>128	1	0.06	1	4
d10-3	32	8	256	32	>128	>128	0.5	0.06	2	4
Δ*nsrR*	0.06	2	128	32	128	128	0.06	0.06	0.06	4
Δ*nsrR-c*	0.125	0.5	16	32	128	128	0.125	0.06	0.06	2
Δ*ndh*	0.06	2	128	32	128	128	0.125	0.06	0.06	4
Δ*ndh-c*	0.125	0.5	16	32	128	128	0.125	0.06	0.06	2

*The list of abbreviations of antimicrobial agents: TCS, Triclosan; CIP, Ciprofloxacin; FOS, Fosfomycin; TIG, Tigecycline; TET, Tetracycline; CHL, Chloramphenicol; CTZ, Ceftazidime; MERO, Meropenem; CTX, Cefotaxime; COL, Colistin.

Table S5 List of genomic mutations and their predicted effects identified in triclosan-evolved Kp85 clones.

Sample day	Clone ID	Mutation in chromosomal genes							
		hp (OPCLNJD_02482)	*nsrR*	*ndhC**	*ndh*	*fabI*	*spy/astE2*	*rppH**	*nuoC*
day7	d7-1		intergenic (+337/-77)	coding (30/360 nt)	T37P	A21T	Intergenic (-213/+134)		
day7	d7-2	Intergenic (-81/+34)	R61L		S67R			coding	
day7	d7-3				G123D				
day7	d7-4		P7L		K92*				
day7	d7-5	Intergenic (-81/+34)	R61L		S67R			coding	
day8	d8-1		Intergenic (+337/-77)		T37P	A21T			W17*
day8	d8-3	116 mutations							
day8	d8-2		R61L						
day8	d8-4		R61L						
day9	d9-2	Intergenic (-81/+34)	R61L		S67R			coding	
day9	d9-3	72 mutations							
day10	**d10-2#**	Intergenic (-81/+34)	R61L		S67R			coding	
day10	d10-3				G123D				

*INDEL: insertions/deletions of nucleotides, nt-nucleotides. No mutation was indicated as blank. #the evolved strain was selected for further RNAseq analysis.

Table S6 one-way ANOVA analysis of conjugation frequency among TRMs and parental strains for main Fig.2d and Fig.2e

**Method**	**by plating (linked to Fig.2d)**			**by flow (linked to Fig.2e)**		
**Plasmid**	pX3_NDM-5	pX4_MCR-1	pFII_MCR-8	pX4_MCR-1	pFII_MCR-8	pA/C_MCR-8
**F (7, 16)**	14.25	5.409	7.127	31.09	1.19	4.017
P* value	<0.0001	0.0025	0.0006	<0.0001	0.362	0.0101

* the ordinary one-way ANOVA was applied in the statistic analysis

Table S7 The list of 54 *Klebsiella* phages tested in this study and their respective host ranges.

Phage ID	Bacterial genotype					
	Kp85anc	Kp85-d10-1	ΔnsrR	ΔnsrR-c	Δndh	Δndh-c
p40	+++	++++	++++	++++	+++	+++
p8	++	++++	++	+	++	+
p10	++	++++	++	++	++	+
p32	++	+	+	+	++	+
p38	++	++++	+++	++++	+	+
p13	++	++++	+	+	+	+
p75	+	+	++++	++++	++++	+++
p77	+	+	++++	++++	++++	+++
p25	+	++++	++++	++++	++	++
p31	+	++	+++	+++	++	+++
p16	+	++++	+	+	+	+
p36	+	++++	++	++	+	+
p45	+	++++	+++	+++	+	+
p59	+	++	+	+	++	+
p53	+	++++	++++	++++	+	++
p55	+	++++	++++	++++	+	+++
p56	+	++++	++	+++	-	+
p69	+	++++	++++	++++	+	+++
p70	+	++++	++	++	++	+
p72	+	++++	++	++	+	+
p78	+	++++	++	++	-	+
p86	+	+	+	+	+	+
p84	+	++++	+	+	+	+
p100	+	++++	+++	+++	+	+++
p1	+	++++	++++	+++	+	+++
p3	+	++++	+++	+	+	+
p6	+	++++	+	+	+	+
p12	+	++++	+	+	+	+
p15	+	++++	+++	++	+	++
p35	+	++++	+	+	+	+
p49	+	++++	+++	++	+	++
p52	+	++++	+++	+++	+	+
p58	+	++++	++	++	+	+
p61	+	++++	++	++	+	+
p20	+	++	++	++	+	+++
p23	+	++++	++	+	+	+
p28	-	+	-	-	-	-
p30	-	++++	++++	++++	+	+++
p93	-	++	+	+	+	-
p64	-	++	++	+	++	+
p71	-	+	-	-	-	-
p76	-	++	+	+	+	-
p87	-	+++	+	+	-	+
p85	-	++	++	++	-	++
p39	-	+	+	+	-	+
p46	-	++	+	-	-	-
p54	-	+	-	+	-	-
p73	-	++	+	+	+	+
p96	-	++++	+	+	-	-
p22	-	++++	+	-	-	-
p13	-	++++	++++	++++	+	+
p48	-	++++	+++	+	-	+++
p51	-	+++	+	+	-	+
p2	-	+++	-	-	-	-

Note: Variation in phage infectivity are as follows: "+" indicates weak phage infection (a few

individual plaques); "++" medium phage infection (substantial turbidity); "+++" strong phage infection (mostly clearing); "++++" very strong phage infection (complete clearing) or "-" no phage infection (no plaques).

Supplementary Figure S1-S8

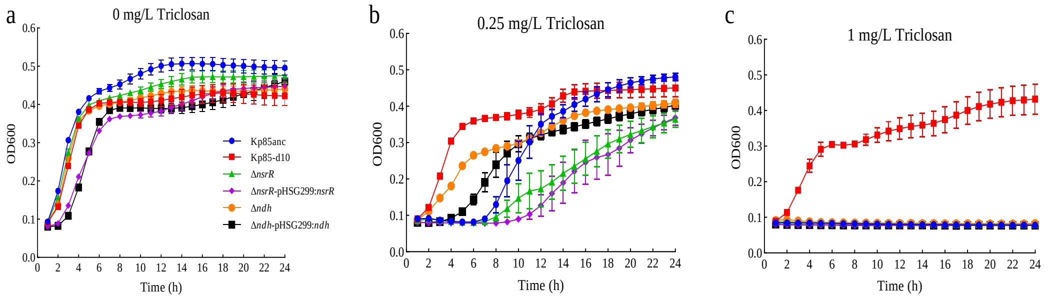

Fig.S1 The physiological effects of *nsrR* and *ndh* genes on bacterial growth in the absence (a) or presence of triclosan (b-c). The deletion of *nsrR* causes detrimental effect on growth rate when treated with 0.25 mg/L triclosan. Except for evolved strain Kp85-d10, no bacterial grew in ancestral or knockout mutants, when treated with 1 mg/L triclosan. Error bars represent the SEM (N=3).

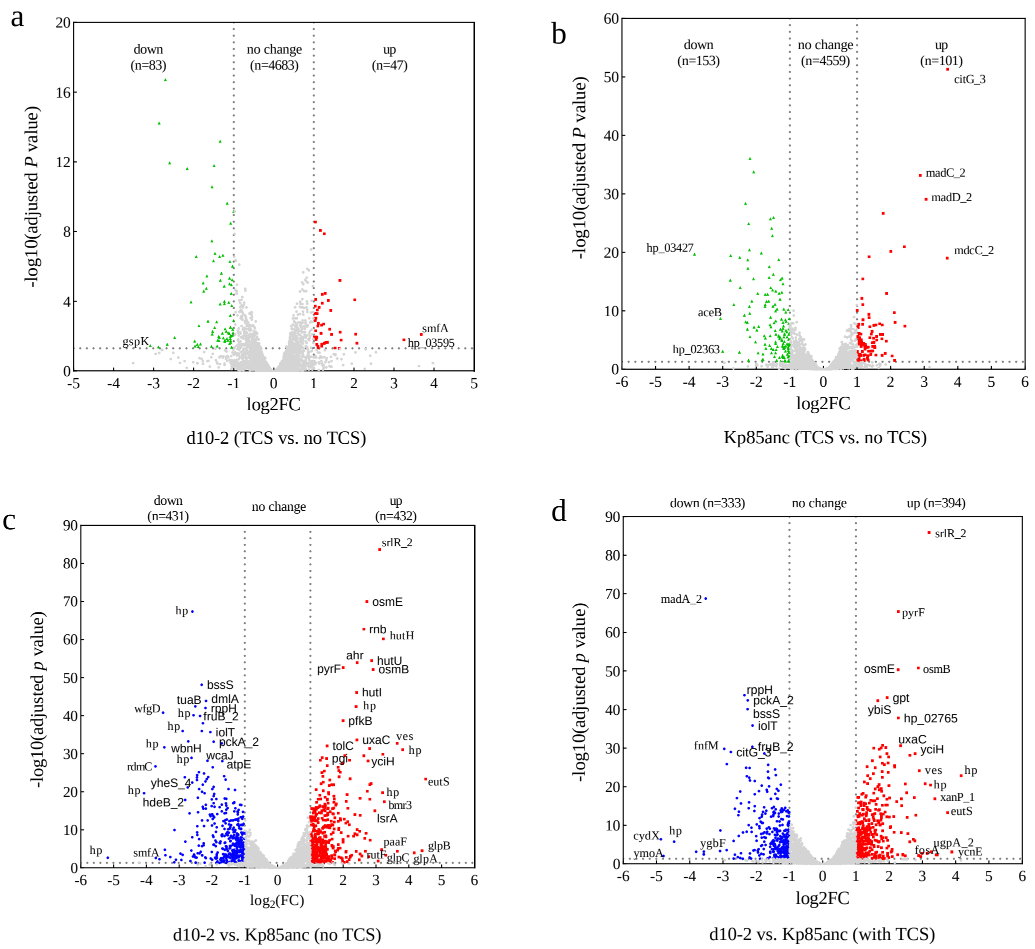

Fig.S2 The gene expression profiles of parental and evolved clone were modulated by TCS exposure. (a) the differential expression of evolved d10-2 in the present of triclosan, relative to that in the absence of triclosan. (b) the differential expression of parental Kp85anc in the present of triclosan, relative to that in the absence of triclosan. (c-d) The differential expression of evolved d10-2 relative to the parental Kp85anc in the absence or presence of triclosan, respectively. Three replicates were conducted for each condition and the data is represented as mean expression of each gene (n=3). The colored areas define genes (dots) with statistically significant differential expression (cut-off setting to adjusted p value less than 0.05 and> 2-fold changes). Blue, red and grey colors represent downregulated (log2(FC)<−1), upregulated (log2(FC)>1) and non-differentially expressed genes, respectively.

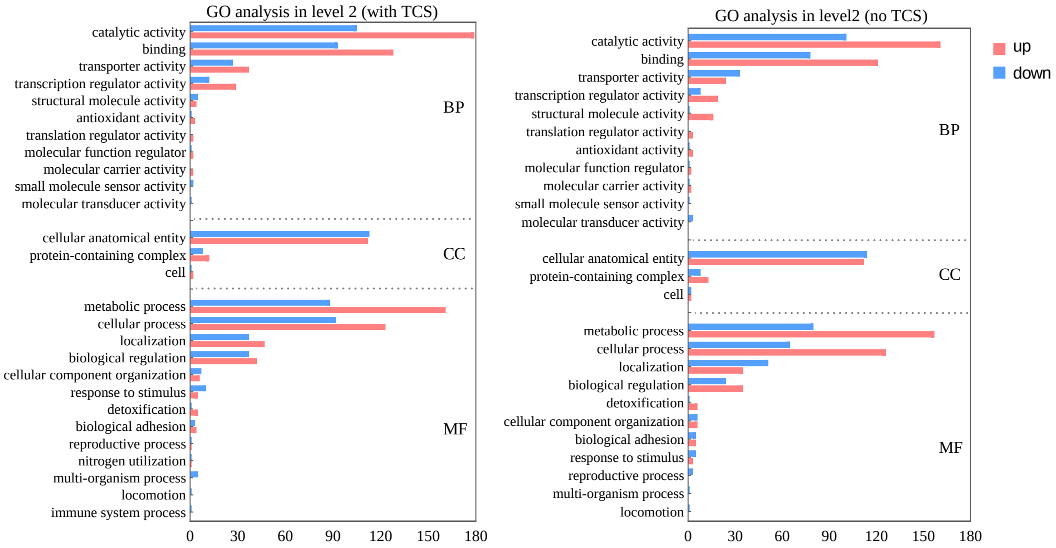

Fig.S3 GO analysis of differentially expressed genes associated with biological processes (BP), cell composition (CC) and molecular function (MF). Both panels show the impact of triclosan resistance on expression of the evolved d10-2 relative to the parental Kp85anc, in the presence (left) or absence (right) of triclosan, respectively.

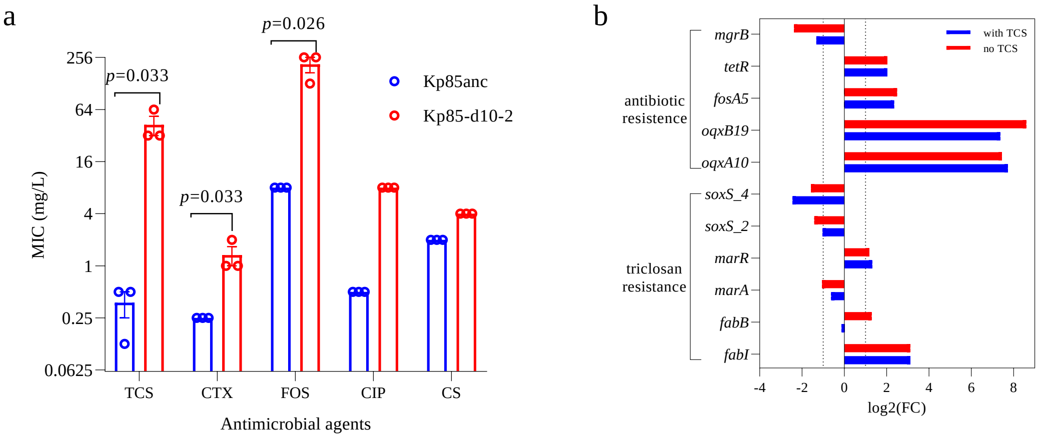

Fig.S4 (a) The multidrug-resistance phenotypes of parental Kp85anc and evolved strain d10-2, determined by agar microdilution to triclosan (TCS), cefotaxime (CTX), Fosfomycin (FOS), ciprofloxacin (CIP) and colistin (CS). **(b)** Triclosan resistance mediates cross-resistance to clinical antibiotics and is associated with clear changes in the expression of triclosan and antibiotic resistance genes, both in the absence (red) and presence of triclosan (blue). All data is based on three independent experiments (mean ± SEM, n=3). The statistical analysis was performed using *t*-test comparing mean differences between each group and *p* values were showed in each bar.

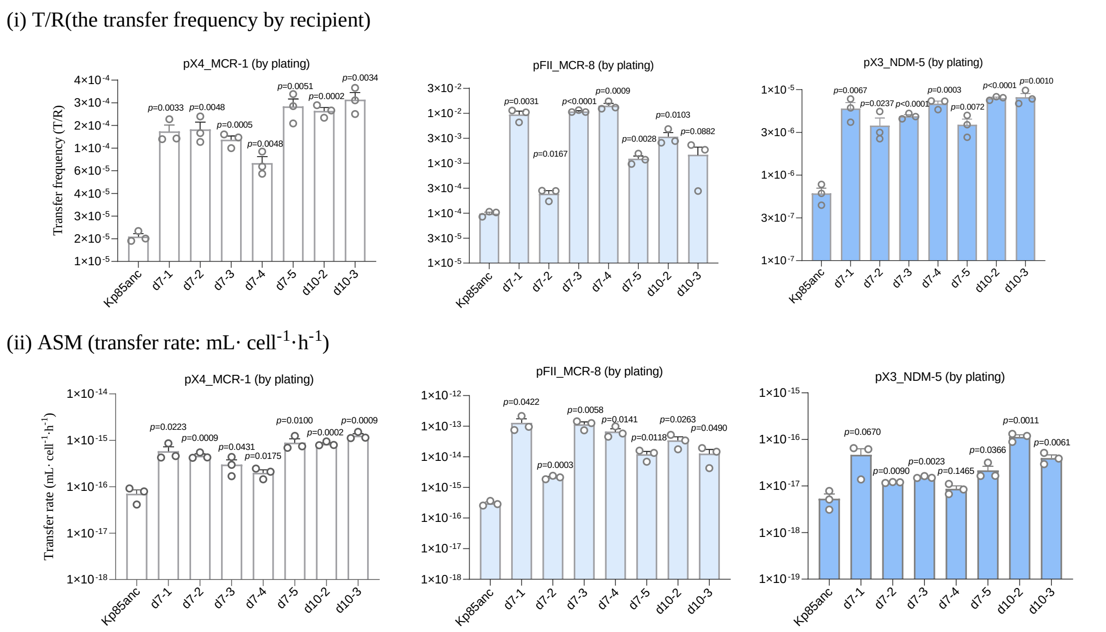

Fig.S5 Conjugation rates were also calculated by two different methods (T/R and ASM). Bacterial densities were measure by selective agar plating. All of these datasets demonstrated that the majority of TRMs have improved conjugation permissiveness. The details for each method are available in Method section. All data were obtained in three independent experiments (n=3). The statistical analysis was performed by Holm-Sidak corrected two sampled *t*-test compared mean differences between parental strain and each TRM strain (Graphpad Prism 8.3.0). The *p*-values were displayed on the corresponding bars. The online calculation data and exact transfer rates were available in Supplementary dataset 1 and 4.

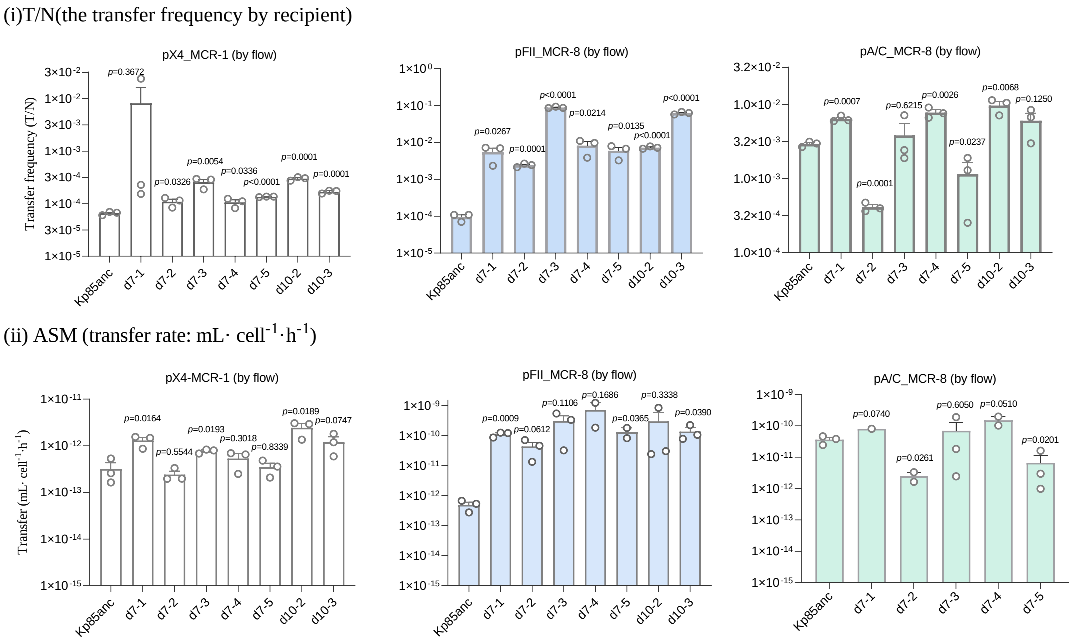

Fig.S6 Conjugation rates were also calculated by three different methods (T/N and ASM). Bacterial densities were measure by flow cytometer. All of these datasets consistently demonstrated that the TRMs have improved conjugation permissiveness. The details for each method are available in Method section. All data were obtained in three independent experiments (n=3). The statistical analysis was performed by Holm-Sidak corrected two sampled *t*-test compared mean differences between parental strain and each TRM strain. The adjusted *p*-values were displayed on the corresponding bars. The online calculation data and exact transfer rates were available in Supplementary dataset 1 and 4. Note that there were some missing replicates in ASM calculations for plasmid pA/C_MCR-8, due to the calculation error presented in online calculation website.

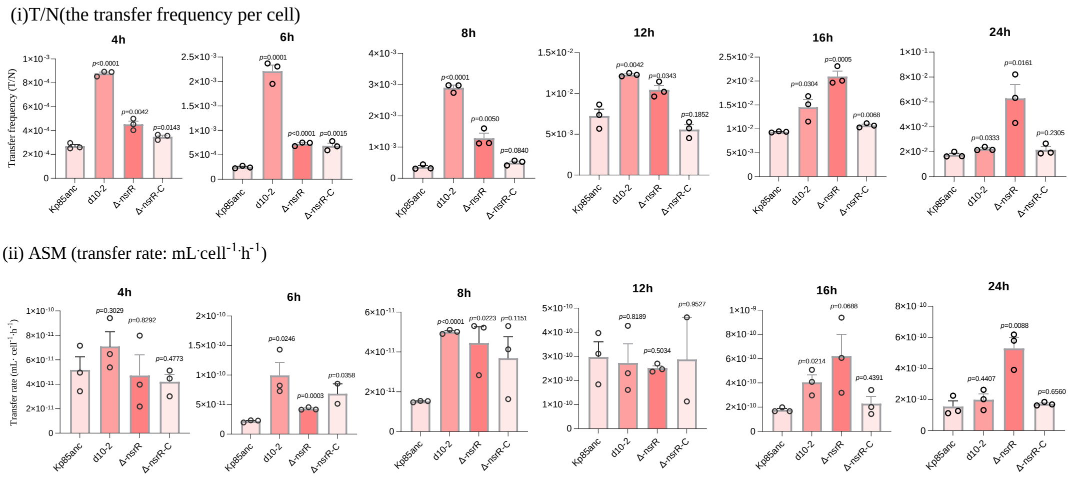

Fig.S7 Conjugation rates of plasmid pFII_MCR-8 were further calculated by two different methods (T/R and ASM). Bacterial densities were measure by flow cytometer. All of these datasets consistently demonstrated that the d10-2 and ΔnsrR strains have improved conjugation permissiveness. The details for each method are available in Method section. All data were obtained in three independent experiments (n=3). The statistical analysis was performed by Holm-Sidak corrected two sampled *t*-test compared mean differences between parental and each *nsrR* variant group. The adjusted *p*-values were displayed on the corresponding bars. The online calculation data and exact transfer rates were available in Supplementary dataset 2 and 4.

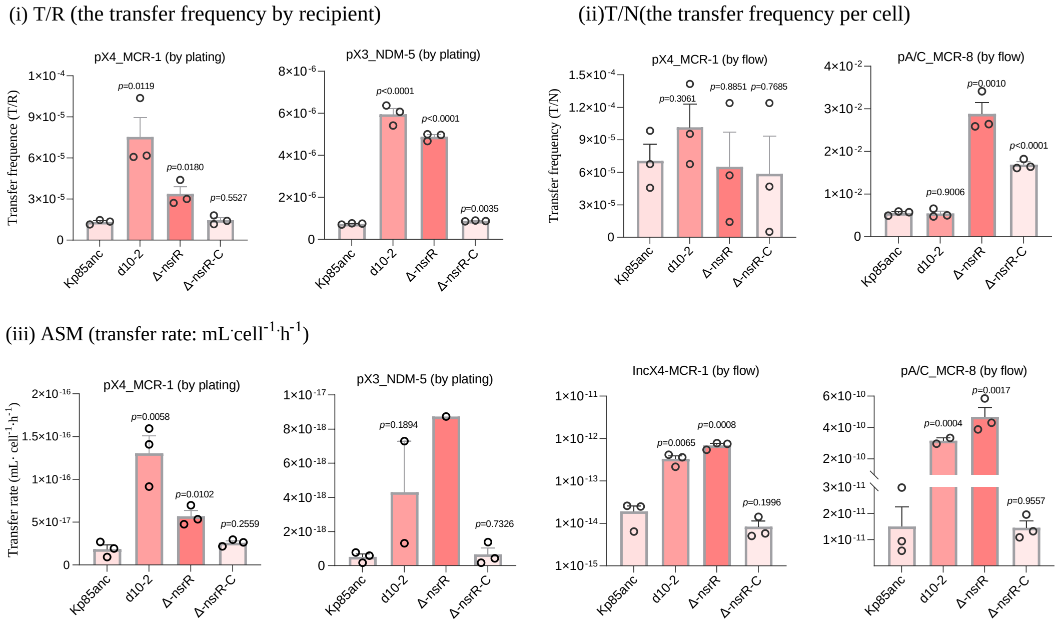

Fig.S8 Conjugation rates of three AMR plasmids were calculated by two different methods (T/N and ASM). Bacterial densities were measure by selective agar plating (c) and flow cytometer (d). All of these datasets consistently demonstrated that the

d10-2 and ΔnsrR strains have improved conjugation permissiveness. The details for each method are available in Method section. All data were obtained in three independent experiments (n=3). The statistical analysis was performed by Holm-Sidak corrected two sampled *t*-test compared mean differences between parental and each *nsrR* variant group. The adjusted *p*-values were displayed on the corresponding bars. The online calculation data and exact transfer rates were available in Supplementary dataset 3 and 4.

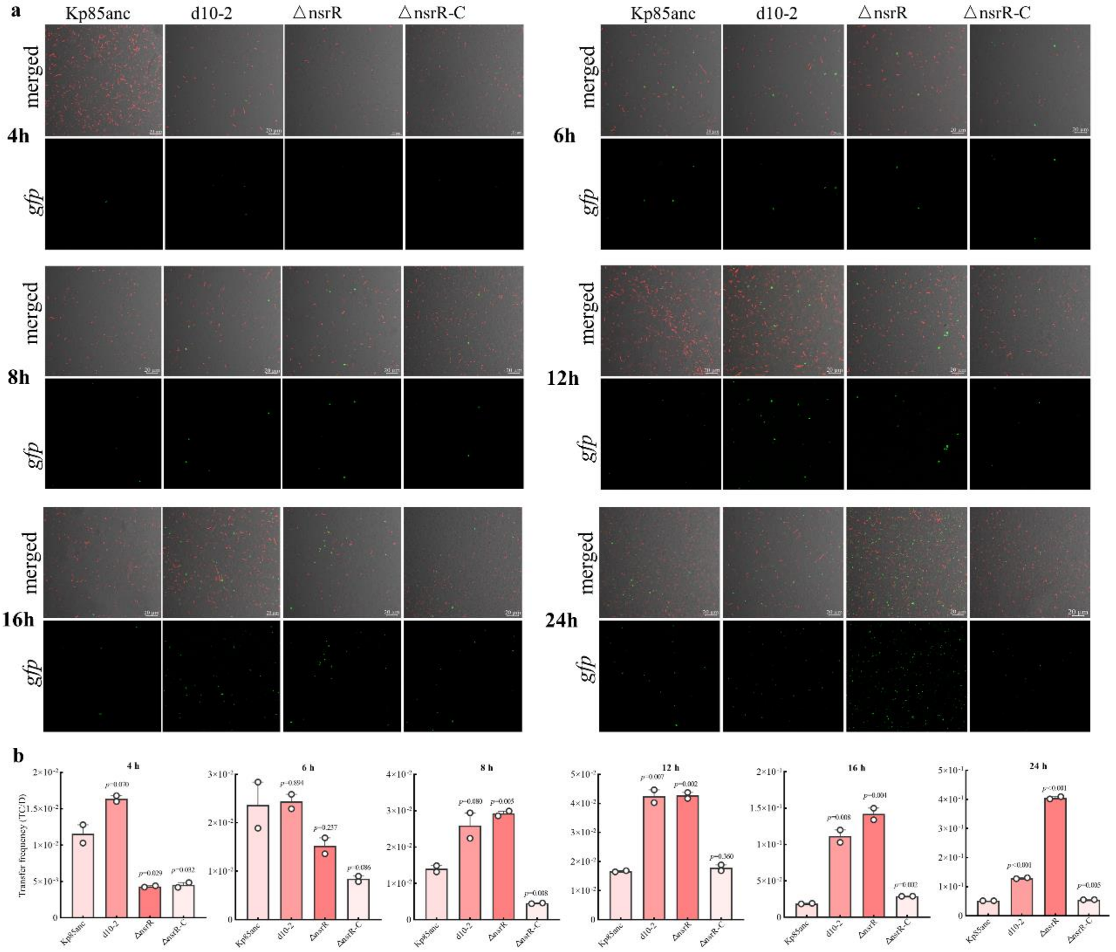

Fig.S9 The increased conjugation rates of AMR plasmids were repeatedly observed in evolved and Δ*nsrR* knockout clones. Panels (**a**) Time-course showing of the dynamics of plasmid transfer (pFII-MCR-8) visualized via confocal laser scanning microscopy and flow cytometry (showed in main **Fig.3a**). In microscopy images, transconjugants and donor cells are shown on green and red colors, respectively, with scale bar of 20 µm. Two independent replicates were performed (n=2). (**b**) The conjugation rates were calculated by dividing the number of *gfp*-expressing transconjugants to the number of *mCherry*-positive donor strain, using ImageJ. The statistical analysis using unpaired *t*-test, and $p<0.05$ denotes statistical significance (n=2).

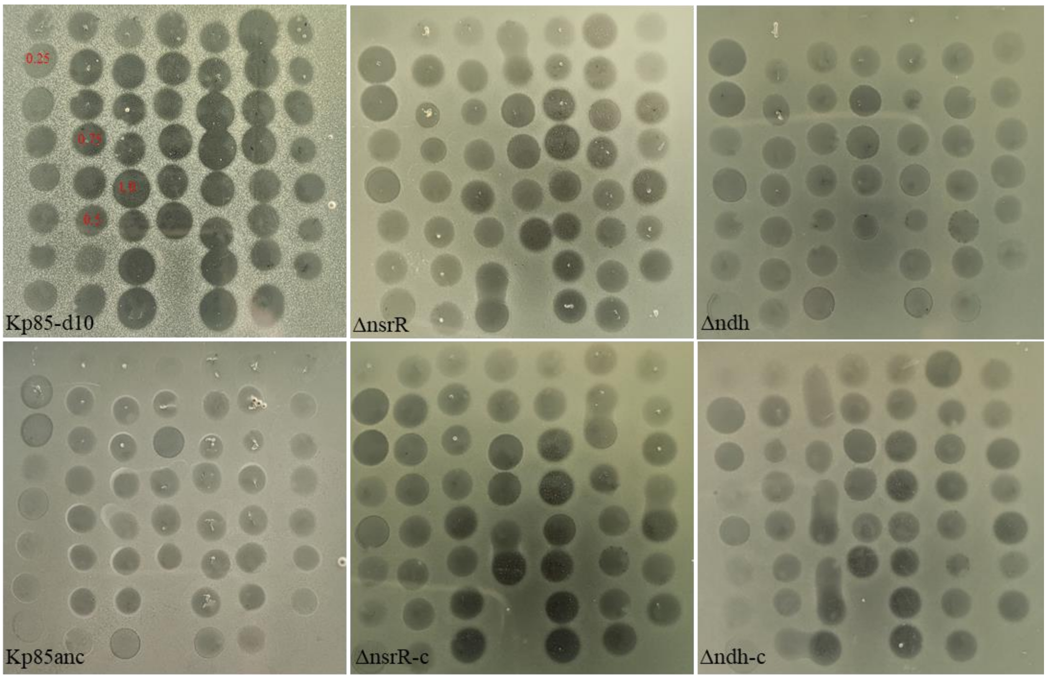

Fig.S10 The phage plaque morphologies of six Kp85 genotypes against 53 various *Klebsiella*-specific phages isolated from urban wastewater treatment plants. Phage susceptibility was assessed by spot-test assays. Phage susceptibility was assessed by the plaque clarity based on spot-testing (ranging from 0=no visible infection to 1=fully clear plaques with no growth of resistant colonies). Regarding the results interpretation, we attributed different values based on the clarity and transparency of the plaques obtained after each spot test to represent the results by heat map: (*i*) complete clearing (defined value of 1.00); (*ii*) clearing throughout but with faintly hazy background (defined value of 0.75); (*iii*) substantial turbidity throughout the cleared zone (defined value of 0.5); (*iv*) a few individual plaques (defined value of 0.25); (*v*) no plaques (defined value of 0.00).. The strain ID was indicated in the bottom of each picture, namely ancestral Kp85anc, evolved Kp85-d10-2, knockout Δ*nsrR* and Δ*ndh*, and their respective complementary strains, Δ*nsrR-c* and Δ*ndh-c*.

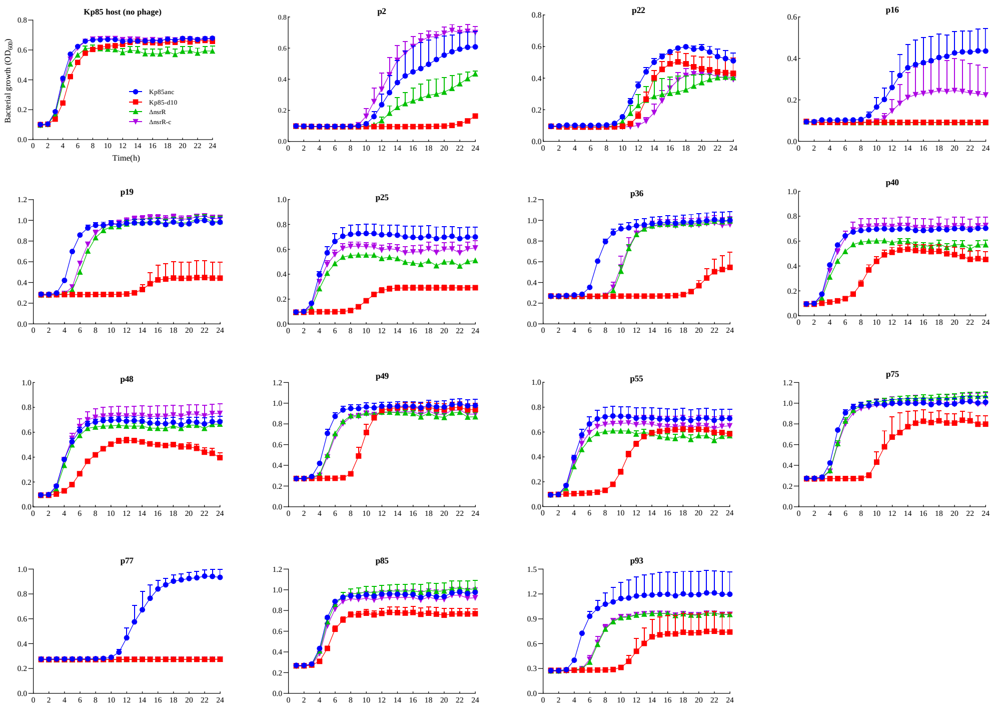

Fig.S11 The growth kinetics of four Kp85 genotypes in the absence (top left panels) and presence of 14 different phages (rest of the panels; phage ID is denoted on top of each panel) (n=14). Data is based on three independent experiments (mean ± SEM, n=3). Blue, red, green and purple colors represent parental Kp85anc, evolved d10-2, ΔnsrR and nsrR-c, respectively.

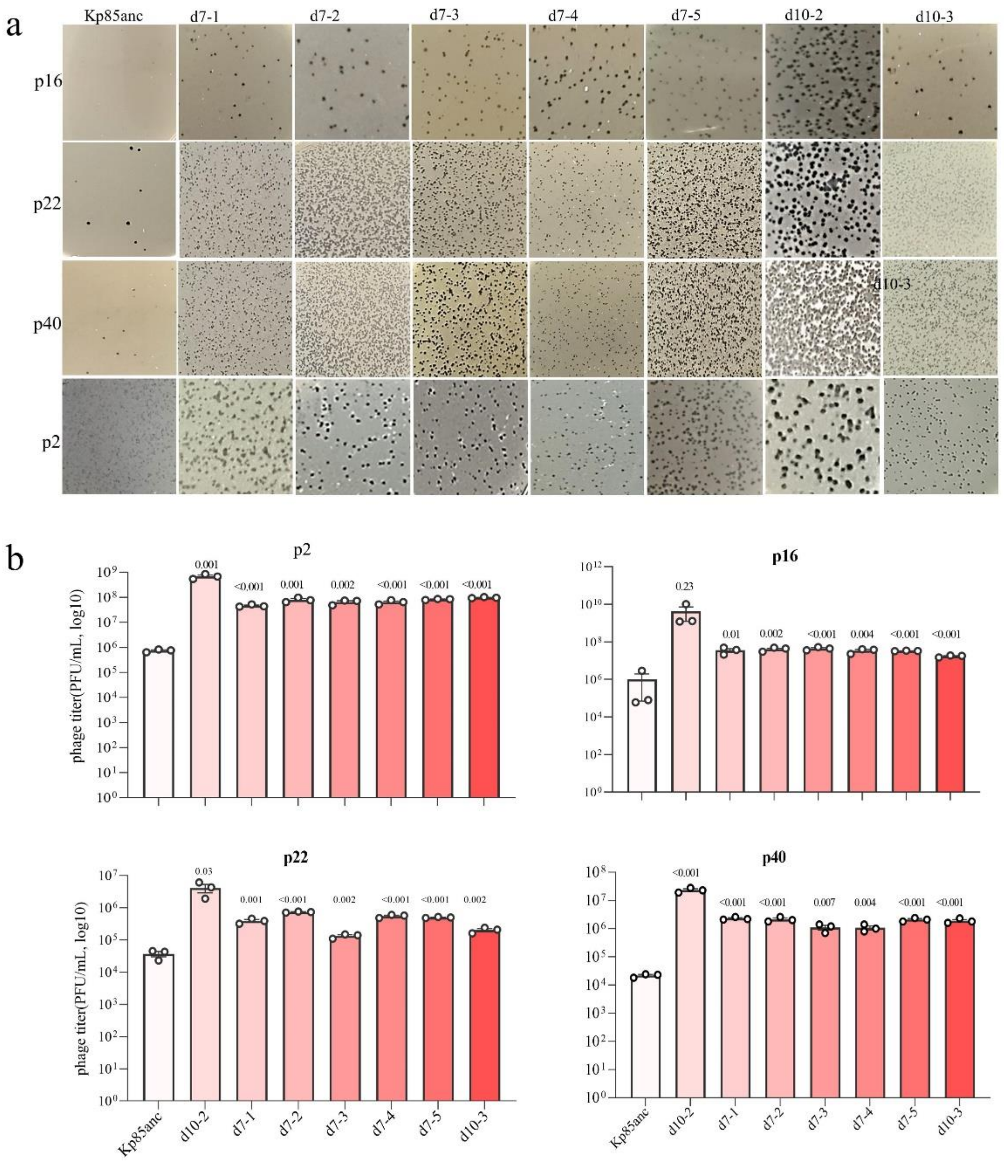

Fig.S12 (**a**) plaque morphologies and numbers of four selected phages in Kp85 variants. The images were selected under the same dilution factor, and a higher numbers of phage plaques were constantly observed in evolved and ΔnsrR strains, indicating the increased phage infectivity in both strains. (**b**) The efficiency of plaque formation of four selected phages (P2, P16, P22 and P40) against seven Kp85 triclosan resistant mutants (TRMs), determining by double-layer agar plate method. All data is based on three independent experiments (mean ± SEM, n=3). The statistical analysis was performed using multiple *t*-test comparing mean differences between each group and adjusted *p* values were showed in each bar.

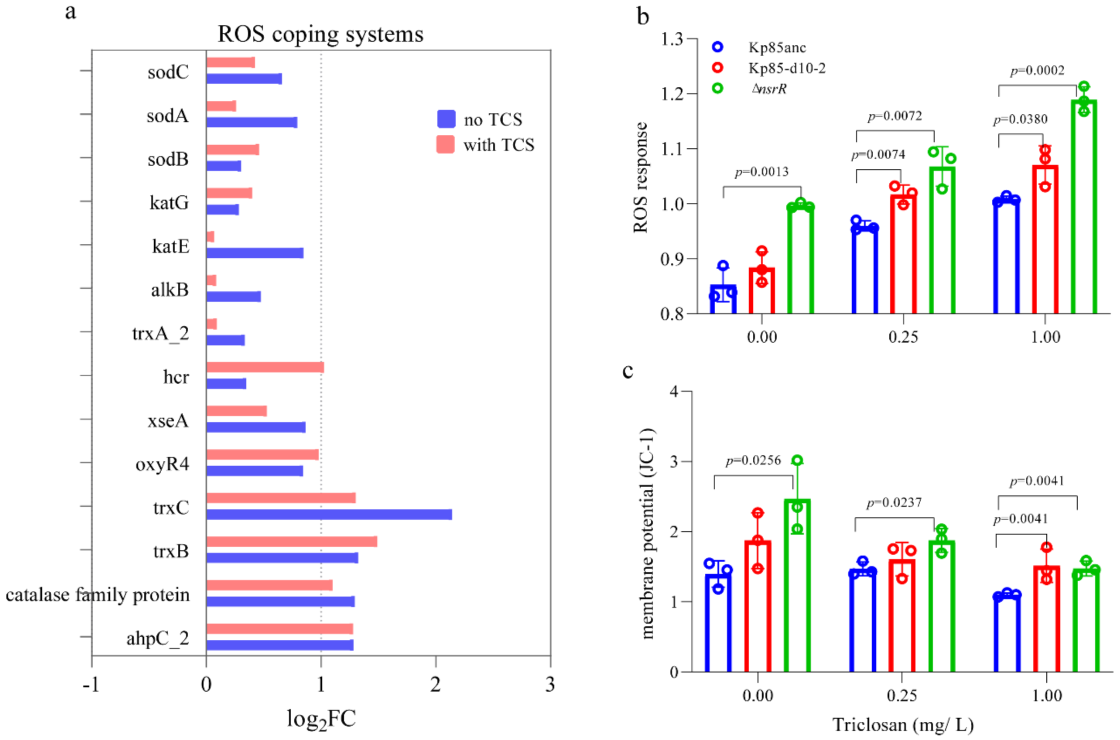

Fig.S13 The impact of *nsrR* gene on ROS response and cell membrane potential. (a) The differential expression of ROS regulation genes in the evolved Kp85-d10-2 relative to the parental Kp85anc in the absence/presence of triclosan. Three replicates were conducted for each condition and the data is represented as mean expression of each gene (n=3). The dotted line was used to define genes with statistically significant differential expression (cut-off setting to adjusted *p* value less than 0.05 and> 2-fold changes). (b) ROS response was compared between three recipient clones in the absence and presence of triclosan. (c) Changes in cell membrane potential measured by JC-1 dye between three recipient clones in the absence and presence of triclosan. In both panels, data is based on three independent experiments (mean ± SEM, n=3) and *p* values were noted in the figure.

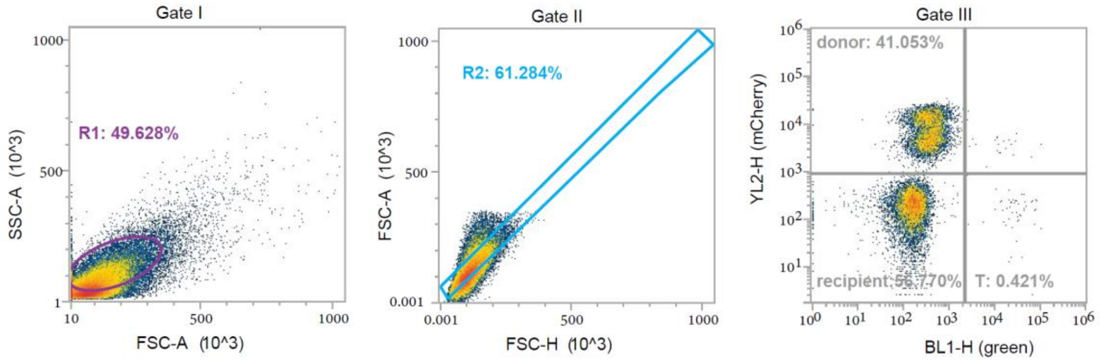

Fig. S14 Gating strategy for analysis of transconjugal cells in mating mixtures by flow cytometry. The procedure consists in three successive gates: Gate I sorts for bacterial size based on forward and side scatter (FSC-A and SSC-A); Gate II identifies single cells using FSC-H versus FSC-A; and Gate III differentiates D, T, R cells, those green cells display red, green or no fluorescence, respectively.

Reference

1 Weishuai Zhai, Y. T., Mi Lu, Muchen Zhang, Huangwei Song, Yulin Fu, Tengfei Ma, Chengtao Sun, Li Bai, Yang Wang, Dejun Liu, Ying Zhang. Presence of Mobile Tigecycline Resistance Gene tet(X4) in Clinical Klebsiella pneumoniae. *Microbiology Spectrum* **10**, e01081-01021 (2021).

2 Ma, T. *et al.* Fitness Cost of blaNDM-5-Carrying p3R-IncX3 Plasmids in Wild-Type NDM-Free Enterobacteriaceae. *Microorganisms* **8**, doi:10.3390/microorganisms8030377 (2020).

3 Wu, B. *et al.* Heterogeneity and Diversity of mcr-8 Genetic Context in Chicken-Associated Klebsiella pneumoniae. *Antimicrob Agents Chemother* **65**, doi:10.1128/AAC.01872-20 (2020).

4 Yang, Q. E. *et al.* Environmental dissemination of mcr-1 positive Enterobacteriaceae by Chrysomya spp. (common blowfly): An increasing public health risk. *Environ Int* **122**, 281-290, doi:10.1016/j.envint.2018.11.021 (2019).

5 Yu Wang, a. S. W., b Weizhong Chen, a Liqiang Song, a Yifei Zhang, a Zhen Shen, c Fangyou Yu, d Min Li, c & Ji, Q. CRISPR-Cas9 and CRISPR-Assisted Cytidine Deaminase Enable Precise and Efficient Genome Editing in Klebsiella pneumoniae. *Appl Environ Microbiol* **84**, e01834-01818. , doi:10.1128/AEM (2018).
