# Supplementary material for: Evolution of triclosan resistance modulates bacterial permissiveness to multidrug resistance plasmids and phages
Source: Nat Commun. 2024 Apr 30;15:3654. doi: 10.1038/s41467-024-48006-9 (PMC11061290; doi:10.1038/s41467-024-48006-9)
Supplement: Supplementary file 3 — Reporting Summary [file 41467_2024_48006_MOESM3_ESM.pdf]

nature portfolio

Corresponding author(s): Timothy R. Walsh *and* Shungui Zhou

Last updated by author(s): 2024-03-14

Reporting Summary

Nature Portfolio wishes to improve the reproducibility of the work that we publish. This form provides structure for consistency and transparency in reporting. For further information on Nature Portfolio policies, see our Editorial Policies and the Editorial Policy Checklist.

Statistics

For all statistical analyses, confirm that the following items are present in the figure legend, table legend, main text, or Methods section.

n/a	Confirmed	
☐	☒	The exact sample size (*n*) for each experimental group/condition, given as a discrete number and unit of measurement
☐	☒	A statement on whether measurements were taken from distinct samples or whether the same sample was measured repeatedly
☐	☒	The statistical test(s) used AND whether they are one- or two-sided<br>*Only common tests should be described solely by name; describe more complex techniques in the Methods section.*
☐	☒	A description of all covariates tested
☐	☒	A description of any assumptions or corrections, such as tests of normality and adjustment for multiple comparisons
☐	☒	A full description of the statistical parameters including central tendency (e.g. means) or other basic estimates (e.g. regression coefficient) AND variation (e.g. standard deviation) or associated estimates of uncertainty (e.g. confidence intervals)
☐	☒	For null hypothesis testing, the test statistic (e.g. *F*, *t*, *r*) with confidence intervals, effect sizes, degrees of freedom and *P* value noted<br>*Give P values as exact values whenever suitable.*
☒	☐	For Bayesian analysis, information on the choice of priors and Markov chain Monte Carlo settings
☒	☐	For hierarchical and complex designs, identification of the appropriate level for tests and full reporting of outcomes
☒	☐	Estimates of effect sizes (e.g. Cohen's *d*, Pearson's *r*), indicating how they were calculated

Our web collection on statistics for biologists contains articles on many of the points above.

Software and code

Policy information about availability of computer code

Data collection	*No software was used for data collection*
Data analysis	Images taken by a confocal laser scanning microscope (CLSM, Leica TCS SP8X DLS, Germany) were visualized by ZEISS ZEN microscopy software. Data analysis was performed using GraphPad Prism (8.3.1). Data shown in plots are represented as mean of three biological independent replicates ± SEM, and exact number of independent replicates for each experiment is stated in their respective figure legends. one/two-way ANOVA or two-tailed t-test analysis ($p < 0.05$) was used to compare differences on bacterial conjugation experiments.

For manuscripts utilizing custom algorithms or software that are central to the research but not yet described in published literature, software must be made available to editors and reviewers. We strongly encourage code deposition in a community repository (e.g. GitHub). See the Nature Portfolio guidelines for submitting code & software for further information.

Data

Policy information about availability of data

All manuscripts must include a data availability statement. This statement should provide the following information, where applicable:

- Accession codes, unique identifiers, or web links for publicly available datasets
- A description of any restrictions on data availability
- For clinical datasets or third party data, please ensure that the statement adheres to our policy

The DNA and RNA-seq sequencing data are available in the NCBI sequence Read Archive With Genbank accession numbers.

The accession numbers for DNA data: (BioProject: PRJNA937637, and BioSample accession No. SAMN33604226 to SAMN33604226). The accession number for RNA-seq data: (BioProject: PRJNA932187, and BioSample accession No. SRR23356413 to SRR23356423). Source dataset are provided in the paper.

Human research participants

Policy information about

Reporting on sex and gender	NA
Population characteristics	NA
Recruitment	NA
Ethics oversight	NA

Note that full information on the approval of the study protocol must also be provided in the manuscript.

Field-specific reporting

Please select the one below that is the best fit for your research. If you are not sure, read the appropriate sections before making your selection.

☐ Life sciences ☐ Behavioural & social sciences ☒ Ecological, evolutionary & environmental sciences

For a reference copy of the document with all sections, see

Ecological, evolutionary & environmental sciences study design

All studies must disclose on these points even when the disclosure is negative.

Study description	for the “evolutionary ramp” selection experiment, one K.pneumoniae was selected and challenged with daily increasing concentrations of triclosan from 0.03 mg/L to 32 mg/L. a total of 15 independent replicates was performed and bacterial density was measured until all replicate population failed to grow. Then we combined whole genome sequencing, reverse genetic engineering, and RNA-seq to test if evolution of chromosomal antimicrobial resistance to triclosan disinfectant has correlated effects on Klebsiella pneumoniae bacterial pathogen permissiveness to MDR plasmids and phage susceptibility.
Research sample	in order to provide the evidence of parallel evolution in TRMs, five independent clones originated from different replicate at day 7 and two TRM clones from day 10 were isolated for further study. the sample selection was also provided in supplementary Table S3
Sampling strategy	A triclosan sensitive (MIC 0.5 mg/L) clinical K. pneumoniae Kp85 strain (Kp85anc) isolated from a female patient was serially cultured on a 96-well plate for 11 days with increasing concentrations of triclosan until all replicate populations (N=15) went extinct. To investigate the genetic mechanisms of resistance, ancestral and evolved clones were isolated from surviving replicates, followed by whole genome sequence. the sample details were also provided in supplementary table S3.
Data collection	strain Kp85anc was serially cultured on a 96-well plate for 11 days with increasing concentrations of triclosan until all replicate populations (N=15) went extinct. and bacterial growth density were measured by Microplate reader(SpectraMax iD3)
Timing and spatial scale	11 days' evolutionary experiment approximately from 15-11-2021 to 25-11-2021, and bacterial density was measured every day
Data exclusions	no data was excluded in this study.
Reproducibility	all experiments were performed at least with three biological replicates, and state all attempts to repeat the experiment were successful.
Randomization	to study the parallel evolution, only one representative clone was selected from each survival replication, see the supplementary table S1
Blinding	The blinding was not relevant to this study, as this study only involved one clinical pathogen and its evolved mutants

Did the study involve field work? ☐ Yes ☒ No

Reporting for specific materials, systems and methods

We require information from authors about some types of materials, experimental systems and methods used in many studies. Here, indicate whether each material, system or method listed is relevant to your study. If you are not sure if a list item applies to your research, read the appropriate section before selecting a response.

Materials & experimental systems

n/a	Involved in the study
☒	☐ Antibodies
☒	☐ Eukaryotic cell lines
☒	☐ Palaeontology and archaeology
☒	☐ Animals and other organisms
☒	☐ Clinical data
☒	☐ Dual use research of concern

Methods

n/a	Involved in the study
☒	☐ ChIP-seq
☐	☒ Flow cytometry
☒	☐ MRI-based neuroimaging

Flow Cytometry

Plots

Confirm that:

☒ The axis labels state the marker and fluorochrome used (e.g. CD4-FITC).

☒ The axis scales are clearly visible. Include numbers along axes only for bottom left plot of group (a 'group' is an analysis of identical markers).

☒ All plots are contour plots with outliers or pseudocolor plots.

☒ A numerical value for number of cells or percentage (with statistics) is provided.

Methodology

Sample preparation	For each mating condition, the percentage of gfp-expressing transconjugants (calculated as transfer rate) in mating cultures were were analyzed using Attune NxT flow cytometer (ThermoFisher, USA)
Instrument	Attune NxT flow cytometer (ThermoFisher, USA)
Software	flowjo_v10 software was used for data analysis
Cell population abundance	bacterial culture was diluted to approximately 100-fold and the setting for cell count was 8000 cells/s c, resulting in transfer rates of each plasmid were calculated as the percentage of gfp-expressing transconjugants in the total 50,000 recorded cells.
Gating strategy	Gating and sorting of transconjugants for each mixed sample were performed based on fluorescent signals, the green fluorescent transconjugant cells and red fluorescent donor cells were excited by 488 nm laser and 561 nm laser, respectively. Fluorescent positive controls of E. coli MG1655::mcherry, E.coli ::gfp and no fluorescence recipient strain were prepared to set PMT voltages and appropriate gating. and the gating strategy can be found in supplementary Fig.S14

☒ Tick this box to confirm that a figure exemplifying the gating strategy is provided in the Supplementary Information.
